# Identification and Compensation for D-Dot Measurement System in Transient Electromagnetic Pulse Measurement

**DOI:** 10.3390/s22218538

**Published:** 2022-11-06

**Authors:** Mengzhe Jin, Hao Li, Shanghe Liu

**Affiliations:** 1 Hebei Key Laboratory for Electromagnetic Environmental Effects and Information Processing, Shijiazhuang Tiedao University, Shijiazhuang 050003, China; 2 National Key Laboratory on Electromagnetic Environment Effects, Army Engineering University of PLA, Shijiazhuang 050043, China

**Keywords:** transient electromagnetic pulse measurement, D-dot sensor, system identification, Hammerstein nonlinear model, system compensation, sensor calibration, broadband system, electromagnetic compatibility

## Abstract

The measurement of the transient pulsed electromagnetic (EM) field is essential for analyzing electromagnetic compatibility. Due to their good performance, D-dot sensors, combined with numerical integration computation for signal recovery, are commonly used to measure electromagnetic pulses (EMPs). However, the integration approach is occasionally flawed due to a non-ideal frequency response or noise, causing distortions in the reconstructed signal. In order to better understand the dynamic performance of the sensor, a nonlinear Hammerstein model is employed in the system identification for the sensor with the calibration data collected in the laboratory environment. When identifying the linear component based on the ultra-wideband characteristics of the measured transient pulse, a two-step identification approach with two different pulse excitation modes, low frequency and high frequency, is utilized to conduct the modeling across the entire frequency range. Based on the reliable identification and modeling of the D-dot sensor, a compensation system that corresponds to the nonlinear Hammerstein model has been developed for the practical signal recovery of the incident E-field. After compensation, the dynamic characteristics of the sensor are significantly improved, and the system compensation approach outperforms the integration method in signal recovery for the incident E-field.

## 1. Introduction

Transient electromagnetic (EM) fields are generated in the form of pulses by various types of discharge (such as electrostatic discharge [1], partial discharge [2], pantograph arcing [3], and transmission line corona [4]), lightning [5], high-voltage switching operation [6], and nuclear detonation [7]. These EM pulses have the typical characteristics of a transient pulse signal with a sharp rising edge and an ultra-wide band (UWB); hence, both the time- and frequency-domain measurements pose some difficulties [8,9]. However, as the military, aerospace, transportation, and electronics industries pay increasing attention to electromagnetic interference (EMI) problems, the undistorted measurement of transient EM fields, which serves as the basis of electromagnetic compatibility (EMC) analysis, is becoming more demanding, and related techniques have become a focus of recent research [10,11,12].

Due to the limitations of dynamic range, response time, frequency bandwidth, and other factors, the typical EMC probe or horn antenna is insufficient for EM pulse measurement [13]. Fortunately, a D-dot sensor is capable of meeting these demands, and is regarded as specialized equipment for transient EMI time-domain testing [14]. The fact that its amplitude and phase vary linearly with frequency means that it is easy to reconstruct the waveform of the incident E-field; therefore, D-dot sensors are widely used in the measurement of UWB pulsed E-fields [15,16].

The D-dot sensor is a type of time-derivative E-field sensor whose theoretical foundation is explained in [17]. In this situation, integration computation is a standard method for post-processing the sensor’s output signal, which is implemented by the design of analog hardware (such as a passive integrator) or signal recovery software (such as a digital integration algorithm). Even though there is evidence of some hardware improvements, such as in [18,19,20], the developed EMP measurement still distorts the input pulse signal to various degrees when circuit design details such as impedance matching, parasitic parameters, and other aspects are absent. As a result, the dynamic performance of the test system with a hardware integrator often needs to be further enhanced. Moreover, the circuit design and analog integrator are overly sensitive to temperature and time drift characteristics, resulting in poor accuracy in the low- and high-frequency response. Refs. [21,22] improved the traditional numerical integration method by using the Tustin equation, Al-Alaoui equation, Gauss–Legendre method, and other numerical calculation methods to ensure the high accuracy and linearity of the integral results. According to these studies, the updated digital integrators provide frequency characteristic curves that are more in line with the ideal integral curve. In addition, evolutionary algorithms have become more prominent in the optimization of digital integrators for performance improvement of digital integrators [23]. However, the accuracy of an integral method is susceptible to noise and the degree of imperfection in the differential characteristics of the sensor. In addition, since the D-dot sensor is not a standard differential device from a broadband perspective, direct integration at high or low frequencies is not effective enough. As a result, the precision of calibration, which is affected by the integral error of the recovery signal, continues to impede its practical application [24].

More researchers have investigated measurement compensation techniques for incident signal recovery on an external computer. Ref. [13] developed a deconvolution algorithm based on the maximum a posteriori (MAP) method for waveform recovery from the output signal of a D-Dot sensor that outperforms the integration method in a subsequent laboratory trial, thereby bringing a unique perspective. Using a single-pole integrator, Ref. [25] presented the transfer function for partial correction of instrumental decay in observed EM field waveforms and then expanded it to the situation of undistorted waveform measurement. Nonetheless, the transfer function is derived theoretically, and the equivalent capacitance of the antenna and parasitic parameters of the circuit may directly affect the actual decay time constant of the sensor, which will lead to the inaccuracy of the theoretical model.

The system identification method based on experimental data is used as an efficient way to create a dynamic mathematical model of the measurement system [17,26]. The identified transfer function model can serve as the foundation for the sensor calibration and waveform reconstruction of the transient electromagnetic field, as well as the prediction of the electromagnetic pulse effect. Unfortunately, most of these studies disregard the nonlinearity of the measurement system, and more studies are conducted in the discrete domain rather than in the continuous domain. However, nonlinearity and continuity are the two properties of real-life systems [27]. A loss of modeling accuracy and several numerical problems may emerge from the linearization and discretization of a nonlinear continuous system with unique properties [28].

Furthermore, since the identification models of the measurement system used in these studies were derived from either high-frequency or low-frequency calibration data, they cannot accurately represent the measurement system’s dynamic characteristics over the entire frequency range from low to high frequencies. The choice of an appropriate calibration signal has a major impact on the identification precision because the operating frequency bands and the operational modes of the identified systems are all generated by excitation of the experimental results [26]. In fact, the dynamic calibration experiments, data acquisition and processing, and system identification algorithms used in the broadband measurement system differ from those used in the traditional low-frequency or narrow-band measurement systems in order to obtain a dynamic model in the entire frequency range. Therefore, to fully utilize the dynamic properties of the sensor, an identification process for broadband sensors that considers both low-frequency and high-frequency features is required.

In summary, direct integration does not operate well at high or low frequencies due to the fact that the D-dot sensor is not a conventional differential device for the E-field signal from a broadband perspective. However, the continuous-time nonlinear Hammerstein system identification methodology proposed in this study can accurately capture the sensor’s non-standard differential features and nonlinearity. In addition, the implementation of the two-step identification approach throughout the identification process can increase the identification model’s dynamic range from near DC to GHz. The identification model constructed via the approach above is more representative of the broadband and non-ideal differential characteristics of the D-dot test system. On this basis, the compensation for the nonlinear Hammerstein model can reconstruct the original incident E-field waveform with more precision, and thus accomplish distortion-free measurements of the transient E-field pulse. This work begins by identifying the nonlinear system model of the D-dot sensor used in transient pulsed E-field measurements. Under the framework of the continuous-time Hammerstein identification method, the linear dynamic component and nonlinear static component are identified with quasi-step pulse excitations, respectively. When considering the UWB properties of transient electric field pulses, a two-step identification method employing both high- and low-frequency calibration datasets effectively improves the dynamic characteristics of linear components across the whole frequency band. In the nonlinear static block, a polynomial fitting model is built using the steady-state values of the sensor response output and excitation input, which are collected by the dynamic calibration test in the laboratory. Then, based on the identification and modeling of the D-dot test system, the compensation system of the nonlinear Hammerstein model is created, and the dynamic performance indexes before and after compensation are compared. Lastly, comparing the compensation result to the excitation signal shows that the compensation method can recover the true signal of the incident pulsed E-field.

## 2. System Identification of the Hammerstein Model in Continuous Time

Most of the identification algorithms of the Hammerstein model in continuous time are derived on the basis that the input excitation signal is random white noise or pseudo-random binary sequences (M-sequence). However, the algorithms derived from such signals are not applicable to the related test of pulsed EM fields. Due to its easy implementation, a step signal has become a commonly used type of input excitation in sensor dynamic calibration. Especially when a square pulse generator is used to generate the excitation signal, if the pulse width of the square wave pulse is much larger than the system response time, the square pulse excitation can actually be regarded as step excitation. However, considering the actual output characteristics of the square pulse excitation source, the step signal is more accurately represented as a quasi-step signal with a specific transition process and a corresponding transition time. The quasi-step signal is presented as follows:

(1) $$x(t)=\{\begin{array}{c} f(t),\;0\leq t\leq t_{0}\\ A,\;t\geq t_{0} \end{array}$$

where *f*(*t*) is the transition process of the quasi-step signal, *A* is the amplitude of the steady state, and *t*_0_ represents the end time of the transition stage. The uncertainty of the transition process and transition time of the quasi-step signal introduces a significant amount of inaccuracy into the dynamic calibration of the test system and the determination of model parameters. Therefore, the approach of nonlinear system identification that employs a quasi-step signal as the excitation source requires more research.

The Hammerstein model is a prominent block-oriented paradigm used to express the dynamic nonlinearity of a system, which consists of a static nonlinear component in series with a dynamic linear component. This section provides the direct parameter identification of the Hammerstein model in the continuous domain under the excitation of a quasi-step signal, hence expanding the algorithm’s applicability.

**Figure 1:**
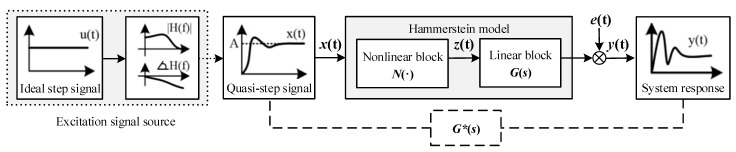
Hammerstein system identification under the excitation of a quasi-step signal.

The block diagram for the implementation of the nonlinear identification method for a single-input single-output (SISO) system is shown in Figure 1. The quasi-step signal *x*(*t*) is the input signal of the system, and *y*(*t*) is the system output signal. *N*(·) and *G*(*s*) are the nonlinear and linear blocks in the Hammerstein model that describes the measurement system, respectively. *z*(*t*) is the intermediate variable between the two blocks, which is approximated by the power series of the input excitation signal *x*(*t*). *e*(*t*) is the noise signal superimposed on the output of the system.

By involving the memoryless polynomial structure in the nonlinear block and the Laplace transfer function of the output error (OE) model in the linear block, the Hammerstein model of the nonlinear system in Figure 1 can be written as follows:

(2) $$\{\begin{array}{c} z(t)=N(x(t))=\sum_{i=1}^{k}c_{i}x^{i}(t)\\ Y(s)=G(s)Z(s)+E(s) \end{array}$$

where i is the polynomial’s order and $c_{i}$ is an arbitrary real number. *X*(*s*), *Y*(*s*), *Z*(*s*), and *E*(*s*) are the Laplace transforms of the system’s input *x*(*t*), output *y*(*t*), intermediate variable *z*(*t*), and noise *e*(*t*), respectively.

*G*(*s*) has the following form:

(3) $$G(s)=\frac{b_{m}s^{m}+b_{m-1}s^{m-1}+\cdots +b_{1}s+b_{0}}{s^{n}+a_{1}s^{n-1}+\cdots +a_{n-1}s+a_{n}},$$

where $a_{1}$, $a_{2}$, …, $a_{n}$, $b_{0}$, $b_{1}$, …, and $b_{m}$ are the parameters of the linear block to be identified. Ref. [29] provided a tutorial introduction for directly identifying linear continuous-time models of dynamic systems from discrete-time sampled data and illustrated the transfer function parameter estimation procedure based on the least squares method.

By substituting Equation (1) for *x*(*t*) in Equation (2), we can obtain the expression of the intermediate variable between nonlinear and linear blocks as follows:

(4) $$z(t)=\sum_{i=1}^{k}c_{i}x^{i}(t)=\{\begin{array}{c} \sum_{i=1}^{k}c_{i}f^{i}(t),\;0\leq t\leq t_{0}\\ h,\;t\geq t_{0} \end{array}.$$

Consequently, *z*(*t*) is likewise a quasi-step signal with the same transition time *t*_0_ as *x*(*t*), and its steady-state value is denoted as *h* with the following calculation method:

(5) $$h=\sum_{i=1}^{k}c_{i}A^{i}.$$

For the linear system G*(*s*) represented by the dashed line in Figure 1, if we use the quasi-step signal *x*(*t*) as the system input and the sensor response signal *y*(*t*) as the system output, we can perform direct linear system identification with the following relational expression:

(6) Y(s)=G*(s)Z(s)+E(s),

where G*(*s*) has the same form as *G*(*s*).

Since the nonlinear element *N*(·) is a product relationship with the linear element *G*(*s*) in the Hammerstein model, it is advisable to set the steady-state gain of *G*(*s*) to 1. As a result, the steady-state gain of the Hammerstein model is represented by the static nonlinear element *N*(·). If the steady-state gain of G*(*s*) is denoted as K*, according to Equation (6), we can obtain the following:

(7) $$y(\infty )=\underset{s\to 0}{\lim\;}sY(s)=\underset{s\to 0}{\lim\;}\;G^{*}\left(s\right)\underset{s\to 0}{\lim\;}\;sX(s)+\underset{s\to 0}{\lim\;}\;sE(s)=K^{*}x(\infty )+e(\infty ).$$

According to Figure 1 and the second row of Equation (2),

(8) $$y(\infty )=\underset{s\to 0}{\lim\;}sY(s)=\underset{s\to 0}{\lim\;}\;G(s)\underset{s\to 0}{\lim\;}\;sZ(s)+\underset{s\to 0}{\lim\;}\;sE(s)=z(\infty )+e(\infty ).$$

By combining and simplifying Equations (7) and (8), and substituting x(∞)=A and $z(\infty )=\sum_{i=1}^{k}c_{i}A^{i}$, we can obtain

(9) $$K^{*}A=\overset{k}{\underset{i=1}{\sum }}c_{i}A^{i}.$$

The matching *N* groups of system output responses *y*(*t*) can be generated by inputting *N* groups of quasi-step signals *x*(*t*) with different steady-state amplitudes *A_j_* (*j* = 1, 2, …, *N*). One transfer function G*(*s*) and one steady-state gain K* can be determined for each pair of input–output signals, and then *N* equations in the pattern of Equation (9) can be obtained as follows:

(10) $$K_{j}^{*}A_{j}=\overset{k}{\underset{i=1}{\sum }}c_{i}A_{j}^{i},\;j=1,\;2,\;\ldots ,\;N.$$

Equation (10) can be written in the form of least squares as follows:

(11) $$\Gamma_{N}=\Phi_{N}c,$$

where

$$\Phi_{N}=\left[\begin{array}{cccc} A_{1} & A_{1}^{2} & \cdots & A_{1}^{k}\\ A_{2} & A_{2}^{2} & \cdots & A_{2}^{k}\\ \vdots & \vdots & \cdots & \vdots \\ A_{N} & A_{N}^{2} & \cdots & A_{N}^{k} \end{array}\right],$$

$$c={\left[c_{1},\;c_{2},\;\cdots ,\;c_{k}\right]}^{T},$$

and

$$\Gamma_{N}={\left[K_{1}^{*}A_{1},\;K_{2}^{*}A_{2},\;\cdots ,\;K_{N}^{*}A_{N}\right]}^{T}.$$

When *N* ≥ *k*, Equation (11) becomes an overdetermined system and may be solved using the least squares approach. In this way, the parameter identification result $c={\left[c_{1},c_{2},\cdots ,c_{k}\right]}^{T}$ of the nonlinear element *N*(·) is determined, and the intermediate variable *z*(*t*) of Hammerstein model in Figure 1 is derived using Equation (2). Then, by taking any set of *z*(*t*) and *y*(*t*), the linear component *G*(*s*) in the Hammerstein model can be identified.

## 3. Two-Step Identification Method for Broadband Measurement System

Since the operational frequency points of the identified system and all of its operating modes are activated throughout the experiment, the selection of input signal significantly impacts the identified system’s reliability and accuracy. The minimum criteria for the input signal to be acceptable are that it must constantly activate the system’s dynamic properties throughout the identification period for the system to be identified.

In the time-domain dynamic calibration of the EM field sensor, the square wave pulse is appropriate for calibrating broadband test systems and is frequently used for modeling broadband systems as excitation signals [8,19,26]. The presence of abundant high-frequency components at the front edge and low-frequency components at the flat top is one of the benefits of square wave excitation signals. When the square wave’s pulse width is greater than the low-frequency response time of the test system, it can be approximated as a step signal, which is also advantageous for implementing the modeling algorithm for the measurement system in Section 2.

For an EM pulse measurement system with a higher upper limit cut-off frequency (*f*_H_), the rise time of the input excitation signal should be shorter than that of the measurable pulse. This ensures that the high-frequency component of the input signal fully covers the upper limit cut-off frequency of the test system, enabling the high-frequency characteristics to be fully activated in the time-domain dynamic calibration. The upper limit cut-off frequency *f*_H_ (in units of Hz) of the identified system is inversely proportional to the rise time *t*_r_ (in units of seconds) of the measurable pulse and can be estimated as follows:

(12) $$f_{H}=\frac{0.35}{t_{r}}.$$

At the same time, since the real EM pulse measurement system often has a particular lower limit cut-off frequency (*f*_L_), it is important to maximize the duration of the input excitation signal in order to fully activate the system’s low-frequency characteristics. This will ensure that the test system’s output response waveform is in a steady state. The lower limit cut-off frequency *f*_L_ (in Hz) of the identified system can be estimated by the flat-top drop δ following the unit step response at time point *t_δ_* (unit is s).

(13) $$f_{L}=\frac{\delta }{{2\pi t}_{\delta }}.$$

**Figure 2:**
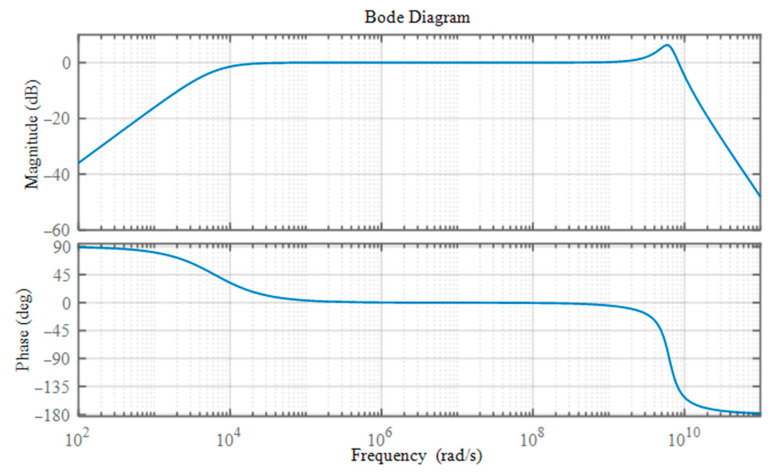
Frequency-response curve of the simulated broadband measurement system.

For the sake of explanation, one can consider a broadband measurement system with a −3 dB operating frequency range from 1 kHz to 1 GHz, which has the following bandpass transfer function:

(14) $$G(s)=G_{H}(s)G_{L}(s)=\frac{s}{s+\omega_{L}}\frac{\omega_{H}^{2}\;}{s^{2}+2\zeta \;s+\omega_{H}^{2}}=\frac{3.95\times 10^{19}s}{s^{3}+3.14\times 10^{9}s^{2}+3.95\times 10^{19}s+2.48\times 10^{23}}$$

where *G*_H_(*s*) and *G*_L_(*s*) are typical first-order high-pass and second-order low-pass filters, and the *G*(*s*)’s lower and upper corner frequencies are *f*_L_ = 1 kHz and *f*_H_ = 1 GHz, respectively. The damping ratio of the low-pass filter is set at *ξ* = 0.25. The frequency response curve of the system is displayed in Figure 2, so that the broadband features of the system in Equation (14) may be observed more clearly.

**Figure 3:**
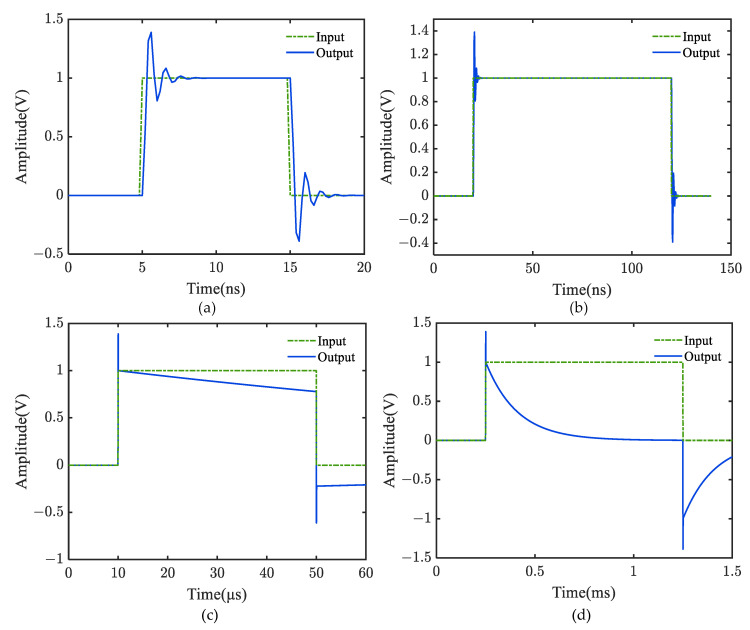
Square wave responses of the simulated broadband measurement system with different pulse widths. (**a**) 10 ns; (**b**) 100 ns; (**c**) 40 µs; (**d**) 1 ms.

The time-domain output response of the broadband measurement system under square wave excitations with different pulse widths is shown in Figure 3, in which the data sampling frequency is set to 5 GHz.

As observed in Figure 3a,b, the high-frequency dynamic features of the system are expressed by ‘ringing’ at the response pulse’s front edge. This phenomenon appears on the frequency characteristic curve as a noticeable resonance peak at high frequency. It should be noticed that the flat-top part of the output response is almost identical to the input, and there is no discernible ‘flat-top fall’. If the identification and modeling solely depend on the two calibration data sets, only the low-pass component *G*_L_(*s*) in *G*(*s*), which reflects the system’s high-frequency response characteristics, can be obtained; however, the measurement system’s low-frequency characteristics cannot be identified.

By extending the pulse width of the input square wave, the measuring system’s low-frequency dynamic properties may be successfully enhanced. Figure 3c shows that even when the pulse width of a square signal is increased to 50 μs, the front edge of the output response pulse still has a noticeable overshoot phenomenon, as shown in Figure 3a,b. In contrast, the output response displays a distinct declining trend in the flat-top region. As shown in Figure 3d, when the square wave’s pulse width increases to 1 ms, the steady-state value of the output response approaches zero, and the flat top fall reaches its maximum value. Since the low-frequency characteristics of the test system are closely related to the flat-top fall of its step response, the excitation signal duration should be increased as much as possible to obtain its step response’s steady-state value.

The system model identified by the calibration data in Figure 3a or Figure 3b may not be similar to the actual measurement model because it can only reflect the high-frequency dynamic response characteristics. To represent the dynamic response characteristics of the measuring system throughout the whole frequency range, the identification of the model should use the calibration data in Figure 3c or even Figure 3d to enhance the low-frequency response characteristics.

However, the hardware requirements for an excitation source that can provide a pulse output with a rise time in the range of hundreds of picoseconds and a duration in the millisecond range or an oscilloscope that can obtain samples continuously for several milliseconds at a sampling frequency of several gigahertz are extremely stringent. In the meantime, the identification algorithm is hindered by the large quantity of sample data. Consequently, utilizing one-time time-domain calibration for dynamic identification throughout the entire frequency spectrum has certain operational constraints.

For this purpose, the EM pulse measurement system can be dynamically calibrated by employing different pulse excitation sources at both low and high frequencies. The identification and modeling issues of the broadband EM pulse measuring system can be resolved by independently developing the low-frequency and high-frequency dynamic models, then combining them to create a full-band identification model.

**Figure 4:**
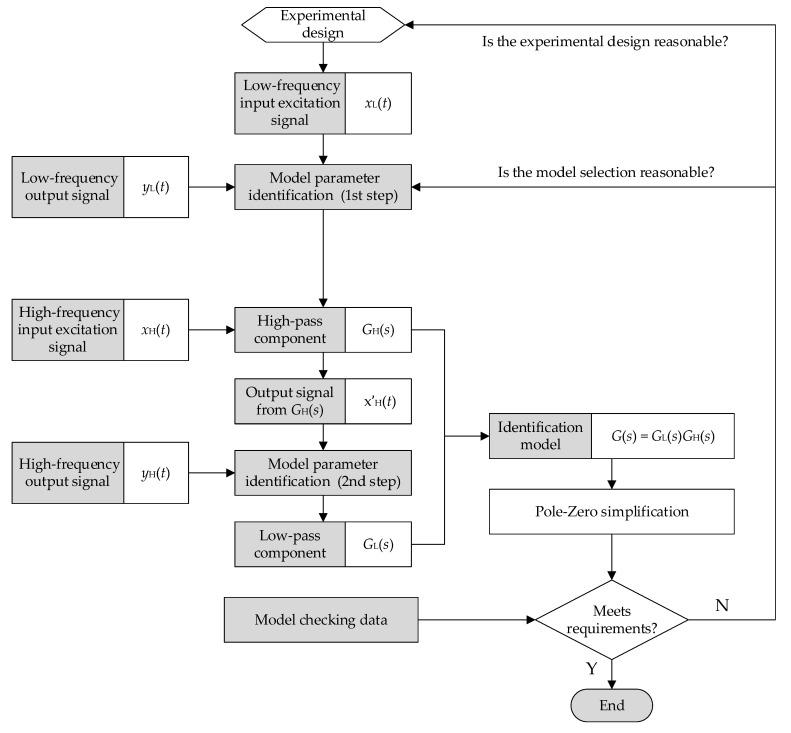
Flowchart of the two-step identification algorithm for broadband measurement system.

The flowchart of the two-step identification algorithm is shown in Figure 4. According to Figure 4, the two-step system identification technique for broadband EM pulse measurement systems can be described as follows:

1. Under the measuring system’s test criteria and the actual working environment, an acceptable time-domain dynamic calibration experiment is created. One must set a low sampling rate to perform low-frequency and long-time sampling on the measurement system to obtain the input and output time-domain signals *x*_L_(*t*) and *y*_L_(*t*); then set a high sampling rate to perform high-frequency and short-time sampling on the measurement system to obtain the input and output time-domain signals *x*_H_(*t*) and *y*_H_(*t*).
2. For a broadband EM pulse measurement system with band-pass characteristics, several octaves often differ in the upper and lower cut-off frequencies. Therefore, it is equivalent to a band-pass filter that consists of a low-pass filter *G*_L_(*s*) and a high-pass filter *G*_H_(*s*), that is, *G*(*s*) = *G*_L_(*s*)*G*_H_(*s*). During the low-frequency calibration process, we select the input excitation signal *x*_L_(*t*) with a long duration and rich low-frequency components, so that the low-pass component *G*_L_(*s*) obtained by the system identification has an all-pass characteristic similar to this low-frequency excitation signal. At the same time, since *G*_L_(*s*) and *G*_H_(*s*) are multiplicative, it can be assumed that the steady-state gain of *G*_L_(*s*) is 1, and then we can obtain the following equation:
  *Y*_L_(*s*) = *G*_L_(*s*)*G*_H_(*s*)*X*_L_(*s*) = *G*_H_(*s*)*X*_L_(*s*) (15)
  Therefore, according to the input signal *x*_L_(*t*) and the output response signal *y*_L_(*t*) of the measurement system, the high-pass component *G*_H_(*s*) of the system can be identified.
3. By filtering the high-frequency signal *x*_H_(*t*) through *G*_H_(*s*) to remove any low-frequency components, the output signal *x′*_H_(*t*) can be obtained, which is employed as the input of the low-pass component in its identification. The equation presented is as follows:
  (16) $$Y_{H}(s)=G_{L}(s)G_{H}(s)X_{H}(s)=G_{L}(s){X^{\prime }}_{H}(s)$$
  In this way, the low-pass component *G*_L_(*s*) of the system can be identified by using *x′*_H_(*t*) and *y*_H_(*t*).
4. Based on *G*_L_(*s*) and *G*_H_(*s*), the identified model of the broadband measurement system is calculated with *G*(*s*) = *G*_L_(*s*)*G*_H_(*s*), and then the model is simplified by canceling the similar zero poles.
5. The identification model is validated with the test data. If the accuracy of the identification model meets the specifications, identification modeling is complete. Or, if there is a considerable difference between the output of the identified model and the actual output of the system, one can review the experimental design, model structure selection, etc., and then return to the appropriate steps to reidentify the system model.

In the identification process of *G*(*s*) and G*(*s*) in the nonlinear Hammerstein model provided in Section 2, the two-step identification approach proposed in this section is used to create the corresponding full-band dynamic model. The derived transfer function is used as the linear block shown in Figure 1 and can more accurately characterize the measurement system.

## 4. D-Dot Sensor Identification and Compensation

### 4.1. Experimental Design

**Figure 5:**
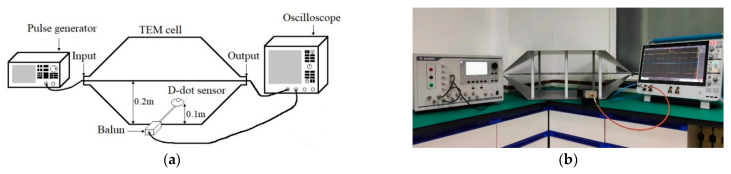
Calibration setup of the D-dot measurement system: (**a**) schematic diagram; (**b**) physical photo.

An experimental setup for the D-dot sensor calibration is developed in the laboratory using a pulse generator, a TEM cell, and a digital storage oscilloscope. The calibration system is constructed with the devices illustrated in Figure 5. The excitation signal generated in the pulse generator is delivered to the TEM cell, and received by the field sensor. The excitation signal and the output signal of the sensor are recorded simultaneously.

The pulse generator can produce square wave pulses with a rise time of 1 ns and pulse width of 1 μs. The square wave pulse is utilized as the excitation source in this experiment, since it possesses both rich high-frequency sharp rising edges and low-frequency flat tops, as mentioned in Section 3. The output end of the pulse generator is connected to the input end of the TEM cell. The pulse excitation signal is fed into the TEM cell to provide a standard pulsed E-field environment. The incident E-field *E*(*t*) inside the TEM cell can be accurately predicted as follows:

(17) $$E(t)=\frac{U\left(t\right)}{d}$$

where *U*(*t*) denotes the excitation voltage applied to the TEM cell and *d* represents the distance between the plates of the TEM cell, which in this experiment was 20 cm. Once the excitation voltage is applied, a homogeneous E-field with approximately transverse mode distribution is formed in the cell. The E-field signals can be detected by the D-dot sensor, whose output end is coupled with a balun and connected to an oscilloscope by a coaxial cable. The Tektronix-MSO64B oscilloscope with a maximum sampling rate of 50 GHz is utilized to collect the excitation voltage signal and the sensor output signal simultaneously.

**Figure 6:**
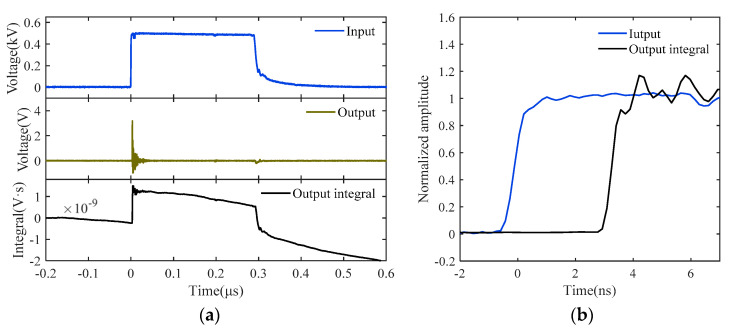
Input excitation, output response, and output integral of D-dot measurement system: (**a**) square wave; (**b**) detailed front edge.

Figure 6 depicts the excitation signal, the sensor’s output signal, and the integrated output response. It is evident that there is a discrepancy between the direct integration result of the output signal and the original excitation signal. Since it can be inferred that the D-dot sensor is not a perfect differential system, it is necessary to construct its system model using the system identification methods given in Section 2 and Section 3. In addition, a generalized cross-correlation phase transform (GCC-PHAT) method [30] is used before each turn of the system identification to remove the delay of the input and output pulse signals, as observed in Figure 6b, which will increase the identification accuracy of the system model.

### 4.2. System Identification for D-Dot Measurement System

The D-dot measuring system has a noticeable differential component, which is in accordance with the D-dot sensor’s operating principle and is depicted in the response waveform in Figure 6. When excited by the quasi-step signal as shown by Equation (1), the differential element will provide a steady-state value of the sensor response output that tends toward zero, which makes it difficult to solve the system’s steady-state gain using the proposed nonlinear identification. In light of this, the method of attaching an integrator in series to the system model is utilized to solve the issue. Specifically, the measured output signal from the identified system is put through the integral element 1/*s* to keep the steady-state response value from becoming zero. After identifying the nonlinear element, a transfer function model with the differential element eliminated is created to characterize the linear component of the measurement system. The model thus determined can be multiplied by *s* to recover the actual system model *G*(*s*) with differential components.

**Figure 7:**
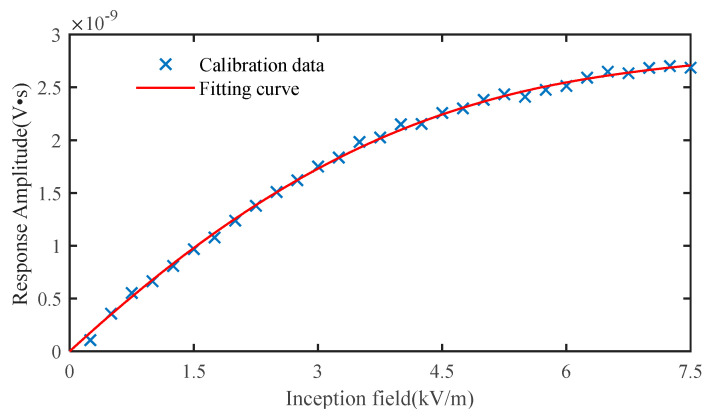
Identification results of the nonlinear element of D-dot sensor.

With the experimental setup, 30 sets of input and output data for time-domain calibration were gathered by varying the amplitude of the square wave voltage throughout the range of 50–1500 V at 50 V intervals. Using the Hammerstein identification approach described in this paper, the identification result for the nonlinear element *N*(·) was first obtained. In the process of solving Equation (11) by least square fitting, the fitting accuracy only marginally improves when the polynomial order k exceeds 4, and the goodness of fit (R-square) was already above 95%. Consequently, the degree of the polynomial in *N*(·) was set to 4. Figure 7 displays the identification results of *N*(·) in the Hammerstein model, wherein the incident field signal serves as input data, and the integrated response serves as output data. The formula for the nonlinear polynomial estimator is as follows:

(18) $$z=3.13\times 10^{-25}x^{4}-3.27\times 10^{-21}x^{3}-4.10\times 10^{-17}x^{2}+7.21\times 10^{-13}x.$$

Equation (2) allows for the production of 30 groups of the intermediate variables *z*(*t*) using the relationship between *z*(*t*) and *x*(*t*). Then, the parameters of the transfer function with the differential element eliminated can be determined using *z*(*t*) as the input and the integration of *y*(*t*) as the output. In this way, the linear element *G*(*s*) in the Hammerstein model can be obtained by multiplying the identified transfer function by the differential element *s*.

Considering the broadband characteristics of the D-dot sensor, we further apply the two-step identification approach outlined in Section 3 to identify the linear block *G*(*s*). Specifically, the low-frequency time-domain calibration data, *x*_L_(*t*) and *y*_L_(*t*), are first acquired at a sampling rate of 250 MHz and a square wave pulse width of 1 μs. Calculated through the nonlinear element *N*(·), the low-frequency intermediate variable *z*_L_(*t*) can be used as the input data in the identification of the low-frequency component, with *y*_L_(*t*) serving as the output. According to the implementation step (2) of the proposed two-step identification method, the high-pass component of *G*(*s*) is obtained as follows:

(19) $$G_{H}\left(s\right)=\frac{3.33\times 10^{6}s}{s^{2}+3.34\times 10^{6}s+2.09\times 10^{10}}$$

Following this, the high-frequency calibration data, *x*_H_(*t*) and *y*_H_(*t*), are collected experimentally with a 12.5 GHz sampling rate and a 50 ns excitation pulse width. Similarly, the high-frequency intermediate variable *z*_H_(*t*) is derived with the nonlinear component *N*(·). According to the implementation step (3), *x’*_H_(*t*) may be first obtained through the high-pass filter and used as the input data for identification. After this, the corresponding low-pass component of *G*(*s*) is identified as follows:

(20) $$G_{L}\left(s\right)=\frac{29.52s^{2}+1.19\times 10^{13}s+3.95\times 10^{10}}{s^{2}+3.14\times 10^{9}s+3.95\times 10^{19}}.$$

Here, five groups of input–output data under different excitation voltages are picked at random to determine the model parameters of *G*_H_(*s*) and *G*_L_(*s*), and the results of the identification process are essentially the same, demonstrating the robustness of the proposed procedure. To further reduce the inaccuracy, the final parameters of *G*(*s*) are determined as the average of the five identification results, and the transfer function form of *G*(*s*) is determined as follows:

(21) $$G\left(s\right)=\frac{9.82\times 10^{7}s^{3}+3.95\times 10^{19}s^{2}+1.31\times 10^{26}s}{s^{4}+3.15\times 10^{9}s^{3}+3.95\times 10^{10}s^{2}+1.32\times 10^{26}s+8.26\times 10^{29}}.$$

**Figure 8:**
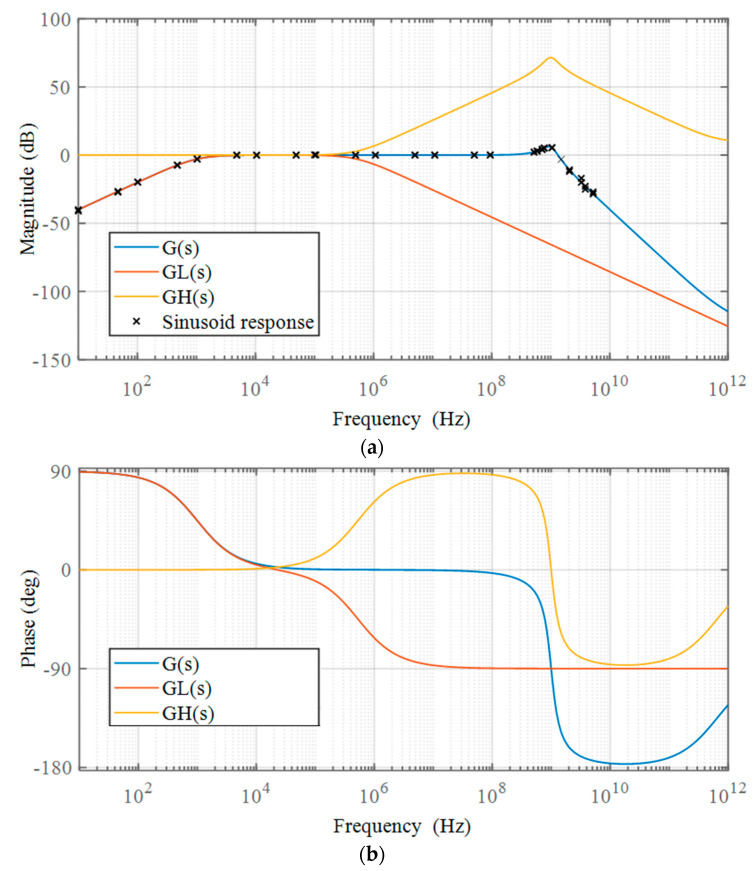
Frequency response for the identified linear component of the D-dot measurement system: (**a**) magnitude response; (**b**) phase response.

As depicted in Figure 8, the frequency-response curve of the identified model for the measurement system, comprising the high-pass and low-pass components, may be generated using Equations (19)–(21). Additionally, 24 measured sinusoid response data are provided in Figure 8a. The measured frequency response is obtained by applying sinusoids of constant amplitude stepped from 10 Hz to 5 GHz and comparing the output amplitude relative to the input.

The comparison results indicate that the frequency–response curve of the identified model is mostly comparable with the measured data, and it makes up for the lack of the system phase-frequency characteristics provided by the measured results. However, at the upper corner frequency, it can also be observed that there is evidence of inaccuracy between the identified model and the measured results, which is mainly caused by noise interference in the measurement. The accuracy of the dynamic model can be further enhanced by increasing the signal-to-noise ratio of the time-domain dynamic calibration data.

Once the dynamic model of the measurement system has been defined based on Equation (21), the dynamic performance index of the system can be retrieved directly, and a suitable dynamic digital compensation filter may be developed for signal recovery. These cases illustrate the benefits of applying system identification techniques to the analysis of the EM pulse measuring system.

Since the nonlinear element *N*(·) and the linear element *G*(*s*) are multiplied in series in the Hammerstein model, the ‘10^−13^’ contained in the polynomial coefficient of Equation (18) can be multiplied by the identified transfer function *G*(*s*) in Equation (21) to cancel the exponent in the polynomial coefficient, thereby reducing the rounding error introduced by the numerical operation. In the meantime, the numerator part in Equation (21) is multiplied by *s* to recover the actual system model *G*(*s*) with differential components. Thus, the Hammerstein identification model of the D-dot broadband measuring system is obtained as follows:

(22) $$\{\begin{array}{c} z\left(t\right)=3.13\times 10^{-12}x{\left(t\right)}^{4}-3.27\times 10^{-9}x{\left(t\right)}^{3}-4.10\times 10^{-4}x{\left(t\right)}^{2}+7.21x\left(t\right)\\ G\left(s\right)=\frac{9.82\times 10^{-6}s^{4}+3.95\times 10^{6}s^{3}+1.31\times 10^{13}s^{2}}{s^{4}+3.15\times 10^{9}s^{3}+3.95\times 10^{10}s^{2}+1.32\times 10^{26}s+8.26\times 10^{29}} \end{array}.$$

**Figure 9:**
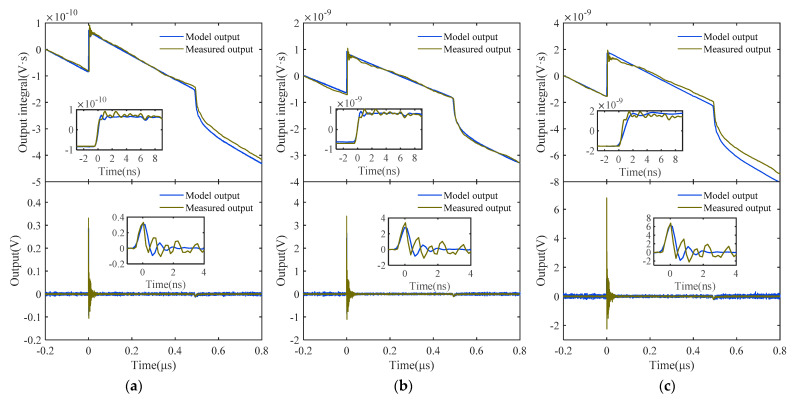
Comparison of the model output with the actual output of the measuring system under different square pulse excitation voltages: (**a**) 50 V; (**b**) 500 V; (**c**) 1500 V.

Figure 9 depicts the comparison between the model output and the measured output at different excitation voltages. In order to demonstrate the efficacy of the algorithm, the calculation results with and without the differential component are shown in Figure 9a–c, respectively.

It can be observed from the graphs that the output of the model corresponds well with the measured output under pulse wave pulses with different amplitudes, which reflects the responsiveness and distortion characteristics of the system under square wave pulse excitation. The goodness of fit between the output of the recognition model and the output of the system under test can still remain above 90% in the presence of noise. The preceding research demonstrates the capability of the suggested identification technique for identifying and modeling the actual EM pulse measuring system in both the frequency and time domains.

### 4.3. Compensation for D-Dot Measurement System and Output Signal Recovery

The observed results in Figure 6 show that even after integration processing, the output signal produced by the D-dot measurement system still exhibits considerable distortion. The true explanation is because the D-dot sensor is not a simple differential system, but rather a nonlinear system with a complex transfer function, as shown in Equation (22). As a result, the dynamic properties of the measurement system, particularly its low-frequency performance, need to be further enhanced. Since the nonlinear identification model of the measuring system is divided into nonlinear and linear components, the corresponding compensation system in this study is also composed of the linear compensation element *G*′(*s*) and nonlinear compensation element *N*′(·). It should be noted that the compensation performed here applies to the identification model shown in Equation (21) that includes a series integral element, rather than the system in Equation (22) that includes the *s* component for differentiation.

Typically, the zero-pole cancellation method is employed to build the linear compensation component [31], and the compensation model structure is created as follows:

(23) $$G^{\prime }\left(s\right)=\frac{1}{G\left(s\right)}\times \frac{\omega_{n}^{2}}{s^{2}+2\xi \omega_{n}s+\omega_{n}^{2}},$$

where ξ and $\omega_{n}$ are the system’s damping ratio and angular frequency, respectively. It is important to keep $\omega_{n}$ at a reasonable value in order to attenuate the high-frequency noise received by the measurement system. With reference to the high-frequency operating point of the original system, it can be noted that $\omega_{n}=2\pi \times 10^{9}$ rad/s in this case. At the same time, ξ is set to be 1 to speed up the system’s response. The linear compensation factor *G*′(*s*) is obtained by substituting the aforementioned parameters into Equation (23).

In order to evaluate the improvement of the sensor’s dynamic performance by the compensation filter, Table 1 gives the comparison of the sensor’s dynamic properties before and after compensation, where *t*_r_, *t*_p_, and *t*_resp_ are the rise time (10–90%), peak time and response time (98% of steady-state value), respectively, and $\omega_{L}$, $\omega_{H}$, and $\omega_{b}$ are the lower cut-off frequency, the upper cut-off frequency, and ± 3dB passband of the sensor, respectively.

All specified dynamic features of the measurement system are greatly enhanced after compensation, as shown in Table 1. The dynamic response time has been decreased dramatically from the hundred-microsecond level to less than one nanosecond, and both the rise time and peak time indicators have also been improved to some extent. In the frequency domain, the passband of the sensor is substantially widened, with the lower cut-off frequency close to DC and the 3 dB frequency band in the high frequency expanded to gigahertz levels.

**Figure 10:**
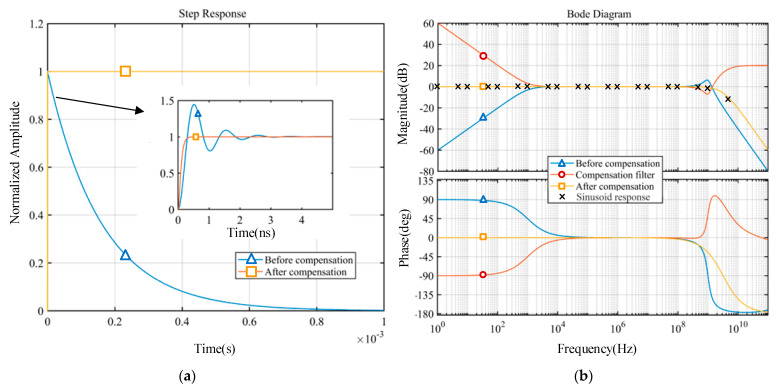
Comparison of the system’s response before and after compensation: (**a**) step response; (**b**) frequency response.

Figure 10 compares the step and frequency response curves before and after dynamic compensation for a more understandable outcome. The compensated system features both low-frequency and high-frequency extensions compared to the original system. Not only did the compensating filter increase the flat bandwidth range, but it also eliminated the 1 GHz high-frequency resonance point. As a result, the ringing in the time domain is no longer noticeable, while the high-frequency noise suppression performance is not far behind. Simultaneously, the sensor’s phase-frequency characteristics have been significantly improved, as depicted in Figure 10b. We further validate the compensating effect using the point representation method depicted in Figure 8a, which involves feeding the measuring sensor with 20 sinusoidal waves of constant amplitude, spanning from 1 Hz to 5 GHz. Equation (23) is then used to correct the sensor output results in order to produce the final reconstructed signal. The frequency response data are obtained by comparing the output amplitude to the input amplitude and are displayed in Figure 10b. Therefore, the experiment results demonstrate that a lower cut-off frequency closer to DC is achievable after compensation.

In addition to linear compensation, nonlinear compensation must be performed on the nonlinear measurement system defined by the Hammerstein model in order to obtain undistorted test data. The primary role of the nonlinear compensation element *N*′(·) is to conduct nonlinear correction on the system’s steady-state gain, so that the steady-state output value of the measurement system has a 1:1 linear relationship with its steady-state input value. The design of *N*′(·) is actually the inversion process of the nonlinear component *N*(·) in the Hammerstein system. Since *N*(·) is a high-order equation, it is challenging to invert it directly. Therefore, the nonlinear component of the compensation system is constructed by switching the roles of the input and output in the original system, that is, the output of the original system is used as the input of the compensation system, and the input of the original system is used as the output to construct the nonlinear part of the compensation system. The coefficient of the nonlinear element of the compensation system is then determined using the least squares method. The following formula for *N*′(·) is derived for the nonlinear element in Equation (18).

(24) $$y^{\prime }\left(t\right)=4.03\times 10^{38}z^{\prime }{\left(t\right)}^{4}-1.75\times 10^{30}z^{\prime }{\left(t\right)}^{3}+2.77\times 10^{21}z^{\prime }{\left(t\right)}^{2}-6.92\times 10^{10}z^{\prime }\left(t\right)$$

**Figure 11:**
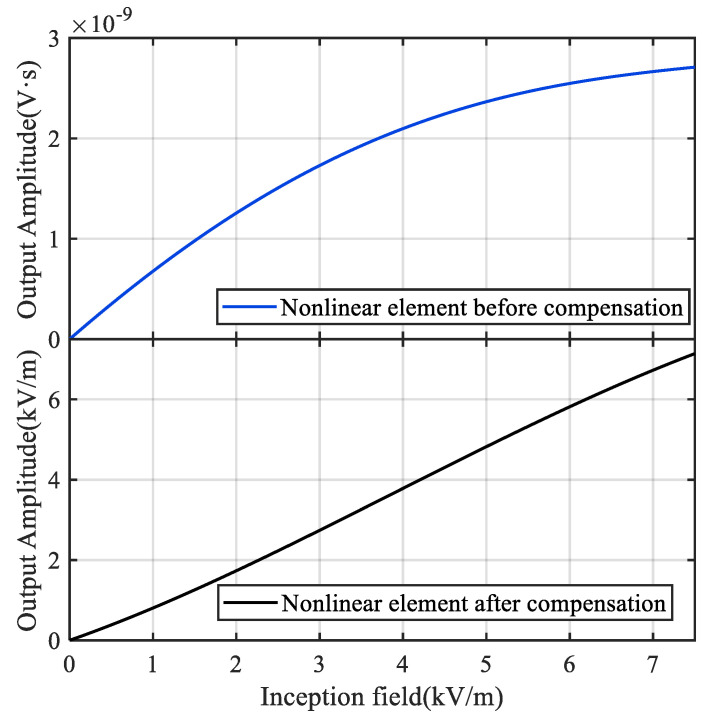
Nonlinear correction of the measurement system.

Figure 11 shows the relationship of the steady-state values between the incident E-field and the sensor’s integrated output before and after nonlinear correction. Following nonlinear compensation, the steady-state output of the sensor is shown in the figure to be in good agreement with the incident field produced in the TEM cell.

**Figure 12:**
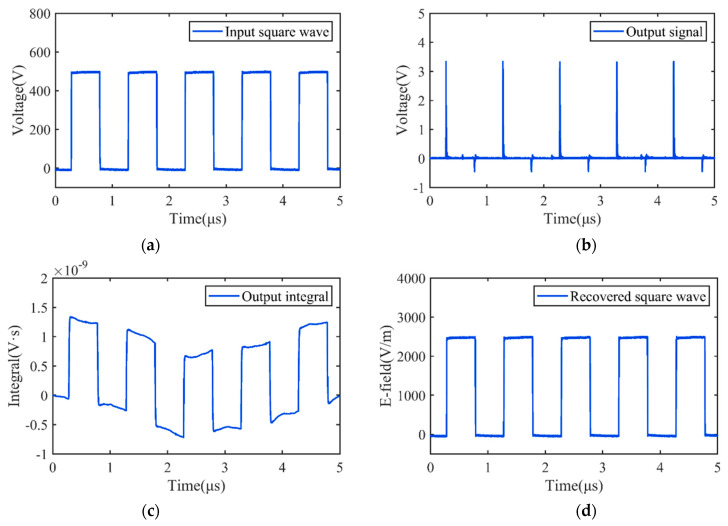
Pulse reconstruction by Hammerstein system compensation.

Figure 12 illustrates the outcomes of reconstructing the measured pulse waveform by making use of the linear and nonlinear compensation components *G*′(*s*) and *N*′(·). This was used to further investigate the practicability of the linear and nonlinear compensation schemes outlined.

The first waveform in Figure 12 is the measured input pulse excitation signal, which consists of five continuous square pulse trains with an amplitude of 0.5 kV. The measured output signals by the sensor and its integral waveform are shown in Figure 12b,c, respectively. Obvious distortions of the square pulse trains can be observed by comparing Figure 12a and Figure 12c, indicating that the direct integration operation is insufficient for the reconstruction of the measured E-field signal. The waveform shown in Figure 12d is the pulse waveform reconstructed from the measured output based on the proposed compensation method, which is in good agreement with the waveform of the sensor’s input voltage signal. Moreover, there is a five-fold relationship between their pulse amplitudes, which is also in good agreement with the theoretical prediction. Consequently, it can be deduced that the proposed modeling and compensating approach can successfully aid in achieving the distortion-free measurement of the transient EM pulse using the D-dot sensor. The dynamic performance of the measuring system is greatly improved after the implementation of compensation, and it has better tracking ability for transient pulse signals, which not only verifies the reliability of the nonlinear Hammerstein model of the pulse field sensor, but also shows the effectiveness of the system identification and the compensation design. The associated methods of Hammerstein nonlinear system identification and compensation described in this study are applicable not only to differential sensors of electromagnetic fields such as D-dot and B-dot, but also to other EM pulse sensors and measurement systems.

## 5. Conclusions

This paper focuses primarily on the continuous-time identification algorithm of the EM pulse measurement system based on discrete experimental data. It analyzes the dynamic properties of the D-dot sensor and its compensation method using the identified mathematical model. The main research results include the following:

(1) A continuous-time identification approach for nonlinear system modeling of electromagnetic pulse measuring systems under quasi-step pulse excitation is suggested based on the Hammerstein model. This method compensates for the inaccuracy of the linear model in the characterization of the actual test system and expands the application of the Hammerstein model in modeling electromagnetic pulse measuring systems.
(2) A two-step strategy for dynamic identification is proposed in light of the difficulty of constructing the mathematical model of the broadband EM pulse measurement system. The method successfully integrates the low-frequency and high-frequency dynamic calibration data collected from a laboratory experiment. The model identified by this method can reveal the dynamic characteristics of the measurement system in the low- to high-frequency range.
(3) With the given identification algorithm, the Hammerstein model of the D-dot sensor for EM pulse measurement is obtained as a combination of a fourth-order polynomial nonlinear element and a fourth-order transfer function linear element. By designing the corresponding compensation system, the lower limit cut-off frequency of the measurement system is improved from kHz to near DC, and the steady-state response time is decreased from μs to ns. The sensor’s nonlinear characteristics are also effectively corrected.

## Figures and Tables

**Table 1:** Comparison of the system’s dynamic properties before and after compensation.

Indicators	Time Domain			Frequency Domain		
	*t*_r_/ns	*t*_p_/ns	*t*_resp_/ns	*ω*_L_/kHz	*ω*_H_/MHz	*ω*_b_/GHz
Before compensation	0.21	0.48	8.16 × 10^5^	1.05	592.94	0.59
After compensation	0.17	0.30	0.28	0.00	2032.64	2.03

## Data Availability

Not applicable.
